# Robot-Assisted Myomectomy for Large Uterine Myomas: A Single Center Experience

**DOI:** 10.1155/2016/4905292

**Published:** 2016-02-29

**Authors:** Vinay Gunnala, Robert Setton, Nigel Pereira, Jian Qun Huang

**Affiliations:** ^1^The Ronald O. Perelman and Claudia Cohen Center for Reproductive Medicine, Weill Cornell Medical College, New York, NY 10021, USA; ^2^Department of Obstetrics and Gynecology, Weill Cornell Medical College, New York, NY 10021, USA; ^3^Department of Obstetrics and Gynecology, Gynecologic Robotic Surgery, NYU Langone Medical Center, New York, NY 10016, USA

## Abstract

*Objective.* To determine if robot-assisted myomectomy (RAM) is feasible for women with large uterine myomas.* Methods.* Retrospective review of one gynecologic surgeon's RAM cases between May 2010 and July 2013. Large uterine myomas, defined as the largest myoma ≥9 cm by preoperative magnetic resonance imaging, was age- and time-matched to controls with the largest myoma <9 cm. Primary surgical outcomes compared were operative time and estimated blood loss (EBL).* Results.* 207 patients were included: 66 (32%) patients were in the ≥9 cm group, while 141 (68%) patients were in the <9 cm group. There was a statistically significant increase in the operative time (130 min versus 92 min) and EBL (100 mL versus 25 mL) for the ≥9 cm group compared to the <9 cm group. Ten (4.8%) patients had the largest myoma measuring ≥15 cm, and 11 (5.3%) patients had a specimen weight >900 gm, of which no major adverse outcomes were observed. All patients in the study cohort were discharged on the same day after surgery.* Conclusion.* RAM is a feasible surgical approach for patients with myomas ≥9 cm. Patients with large myomas undergoing RAM are also candidates for same-day discharge after surgery.

## 1. Introduction

Uterine myomas can cause menorrhagia, dysmenorrhea, pelvic pain, and infertility. Myomectomy, open or minimally invasive, is the preferred surgical modality in patients with symptomatic myomas who desire future childbearing capacity. It is well established that the laparoscopic approach is associated with shorter hospitalization, faster recovery, lesser pain, decreased blood loss, fewer surgical complications, and fewer expenses compared to the abdominal approach [1]. In recent years, as robotic technology has gained momentum in gynecology, studies have emerged indicating that surgical outcomes are similar between traditional laparoscopic and robot-assisted myomectomies [2–4]. In addition to having the same postsurgical benefits, robot-assisted myomectomy (RAM) has the advantage of three-dimensional visualization, improved dexterity, and negation of tremors, which are thought to overcome many of the surgical limitations of traditional laparoscopy. One of the lingering controversies between the two minimally invasive approaches is that, compared to conventional laparoscopy, the robotic approach has been associated with longer operative time and increased expense [2, 3].

Uterine dimensions, myoma size, and location are some of the primary factors used to determine whether an abdominal or minimally invasive surgical approach is utilized. An abdominal approach is often favored with larger, intramural, and posterior myomas, secondary to the difficulties of hemostasis, uterine closure, and tissue removal associated with minimally invasive surgery [5]. Prior studies have demonstrated that myomas measuring up to 8 cm can be safely and effectively removed using a traditional laparoscopic approach [6, 7]. While there is less data regarding RAM, there is evidence suggesting similarities to limits of myoma size compared to laparoscopic surgery [8]. As such, we selected a cutoff of 9 cm or greater to define the “large” myoma group in order to reflect a cohort of patients who may not have previously been considered appropriate candidates for minimally invasive surgery. The primary objective of the current study is to assess the surgical outcomes after RAM for uterine myomas ≥9 cm to determine if it was a feasible approach for women with large myomas. As a secondary objective, we also evaluate the feasibility of same-day discharge after RAM in the same study cohort.

## 2. Materials and Methods

### 2.1. Inclusion and Exclusion Criteria

The New York Hospital Queens institutional review board approved the study protocol. Informed consent by the patients was not required as the study was a retrospective chart review. We reviewed all RAM cases performed by one minimally invasive gynecologic surgeon (JQH) between May 5, 2010, and July 31, 2013. All patients were offered a robot-assisted approach. Other concomitant intraoperative procedures included hysteroscopy, chromotubation, ovarian cystectomy, salpingectomy, salpingo-oophorectomy, and resection of endometriosis. However, all RAM cases included in the study were performed with myomectomy as the primary indication. Patients with intraoperative findings consistent with adenomyosis rather than myomas were excluded.

### 2.2. Operative Technique

All procedures were performed with the patients under general endotracheal anesthesia in the dorsal lithotomy position with adjustable Allen stirrups and lower extremity compression devices for deep vein thrombosis prophylaxis. A VCare® uterine manipulator (CONMED Utica, NY, USA) was placed vaginally. A Veress needle (UNIMAX Taipei, Taiwan) was placed in the umbilicus for insufflation of the peritoneal cavity. A 5 mm bladeless ENDOPATH® XCEL® trocar (Ethicon Endo-Surgery Inc., Cincinnati, Ohio, USA) was introduced into the abdomen using a twisting motion under direct visualization with a 0° 5 mm laparoscope in the left upper quadrant. A 12 mm incision was made intra- or supraumbilically depending on the size of the uterus, through which the operative camera was placed. Three additional 8 mm incisions were made and the robotic trocars were placed under direct visualization. In general, the first 8 mm robotic trocar was placed in the patient's right lower quadrant, 2-3 cm above the anterior superior iliac spine and caudad to the camera port, along an imaginary arc centered at the pubic symphysis. The second 8 mm robotic trocar was placed similarly in the patient's left lower quadrant. The original 5 mm port in the left upper quadrant was used as the assistant port. The patient was placed in the Trendelenburg position and da Vinci robotic surgical system (Intuitive Surgical Inc., Sunnyvale, CA, USA) was side-docked, parallel to the right side of the patient regardless of number or location of myomas. Following careful intraoperative evaluation of the number, position, and size of myomas, dilute vasopressin was injected into the myoma bed for vasoconstriction, in order to minimize the blood loss during surgery. Uterine hysterotomy was made over the prominent bulge of the myoma. Myomas were enucleated intact using Plasma Kinetic (PK) dissecting forceps (Intuitive Surgical Inc., Sunnyvale, CA, USA), EndoWrist tenaculum (Intuitive Surgical Inc., Sunnyvale, CA, USA), and a monopolar cutting device. The uterine defect was repaired with 0 V-Loc suture (Covidien, Mansfield, MA, USA) in at least 2 layers. After closure of the hysterotomy, the abdomen was irrigated and suctioned and then the robot was undocked. The 12 mm incision was extended to 20 mm and the specimen was extracted with electromechanical morcellation. The 20 mm fascial incision was closed with 0 Vicryl*™* suture (Ethicon Endo-Surgery Inc., Cincinnati, Ohio, USA). All skin incisions were injected with bupivacaine hydrochloride and closed with a single interrupted stitch using a 4.0 Biosyn*™* Glycomer*™* 631 suture (Covidien Ltd., Dublin, Ireland). All patients received intravenous ondansetron and dexamethasone for postoperative nausea and ketorolac and hydromorphone for postoperative pain. Patients were discharged home the same day after meeting immediate postoperative milestones (ambulation, pain control, and tolerating liquids orally) and a successful trial of void. Discharge medications included oral ibuprofen and combined acetaminophen/oxycodone.

### 2.3. Outcome Variables Assessed

Demographic characteristics including age, body mass index (kg/m^2^), parity, and prior abdominopelvic surgery of patients meeting inclusion criteria were collected. Preoperative pelvic magnetic resonance imaging (MRI) was used to determine the largest myoma size. In addition, MRI was used to assess myoma characteristics including their location (submucosal, intramural, subserosal, and pedunculated) and number (> or <5 myomas). Total weight of all myomas removed was determined. Histopathologic evaluation of the excised myomas was also performed.

Patients were divided into two groups, a priori, based on the maximum dimension of the largest myoma as determined by preoperative pelvic MRI, that is, ≥9 cm versus <9 cm. The primary surgical outcomes compared between the two groups were estimated blood loss (EBL), operative time (skin-to-skin time), and duration of hospital stay (days). EBL was determined by volume in the suction canister at the end of the procedure and was concurrently agreed upon by the surgical and anesthesia teams. Major adverse outcomes assessed included injury to surrounding organs, conversion to laparotomy, requirement for transfusion of blood products, postoperative infection or abscess formation, and need for hospital readmission. All patients had a standard postoperative follow-up 2 weeks from surgery and again after 2 months.

### 2.4. Statistical Analysis

Data was analyzed using commercially available software (SPSS version 19.0; IBM, Armonk, NY). Student's* t*-test was used for comparison of means, and Mann-Whitney *U* test was used for nonparametric comparisons. In all cases, a *P* value < 0.05 was considered to be statistically significant. The normality of the data was evaluated by examining the skewness and kurtosis of scatter plots and histograms for each of the surgical outcomes.

## 3. Results

Two hundred and seven patients met inclusion criteria during the study period. Of those, 66 (32%) patients had the largest myoma ≥9 cm, while 141 (68%) patients had the largest myoma <9 cm. Demographic characteristics, listed in Table 1, were similar for both groups with the exception that patients in the latter group had significantly more previous abdominal surgery (53% versus 26%, *P* < 0.001). Table 1 also depicts myoma characteristics including number and location. There was no statistical difference between percentages of patients with primarily intramural fibroids and those with >5 myomas. The median specimen weight was 106 gm and 510 gm for the <9 cm and ≥9 cm group, respectively (*P* < 0.001).

Table 2 highlights the primary surgical outcomes assessed in this study. There were significant increases in operative time (130 min versus 92 min, *P* < 0.001) and EBL (100 mL versus 25 mL, *P* < 0.001) for the ≥9 cm group. However, no significant differences in the major adverse outcomes were seen between the two groups. Specifically, the mean hospital stay was zero days: that is, all patients were discharged on the same day after surgery. No RAM cases were converted to laparotomy or had intraoperative injury to surrounding organs. One patient in each group received a blood transfusion. One patient in the <9 cm group developed a postoperative pelvic abscess and was readmitted to the hospital for computed tomographic-assisted drainage and intravenous antibiotics. Of the 207 patients included in the study, 10 (4.8%) patients had the largest myoma measuring ≥15 cm by preoperative imaging, and 11 (5.3%) patients had a specimen weight >900 gm. No adverse surgical outcomes were noted in these groups either. The largest myomas excised during the study period measured 19.8 cm, for which the EBL was 100 mL and operative time was 113 min.

## 4. Discussion

The current study evaluates whether RAM is a feasible and safe surgical approach for large uterine myomas. Results from this study suggest that patients with larger myomas will experience an increase in EBL (100 mL versus 25 mL); however, this difference was not clinically significant, as it did not result in increased rate of blood transfusion. Patients with larger myomas also experienced a longer operative time (130 min versus 92 min). Despite these differences, we did not observe any additional adverse surgical or adverse postoperative outcomes. Furthermore, all patients in the study cohort, including those in the ≥9 cm group, were discharged on the same day of surgery after meeting immediate postoperative milestones.

Previous studies have attempted to establish criteria for candidates to determine whether laparoscopic or RAM would be an effective approach. Given that the role of RAM in gynecologic surgery is still emerging, there is limited literature on its effectiveness and safety profile; therefore, data must be extrapolated from traditional laparoscopy. Of particular concern is the rate of the conversion to laparotomy, in which fibroid size is one of the primary factors that have been shown to play a role. In one study, myomas measuring >5 cm were an independent risk factor related to the risk of conversion to laparotomy using traditional laparoscopy [9]. Conversely, another study of 51 cases with myomas measuring at least 9 cm had no conversions to laparotomy; however, one patient underwent an open hysterectomy hours after surgery due to persistent intra-abdominal hemorrhage [5]. Specific to RAM, a study analyzing 35 RAM cases had 2 cases converted to laparotomy with a conversion rate of 8.6% [10]. The mean myoma diameter was 7.9 ± 3.5 cm (95% CI 6.63–9.13) with the majority of myomas measuring greater than 5 cm [10].

Several studies, including one systematic review and meta-analysis, have also compared other surgical outcomes between abdominal, laparoscopic, and robot-assisted myomectomies [2–4, 11–14]. Studies were controlled in terms of number, diameter, and weight of myomas. Compared to abdominal myomectomy, RAM was associated with significantly lower EBL, decreased rate of blood transfusion (relative risk 0.37; 95% CI, 0.16–0.85), less postoperative fever (relative risk 0.07; 95% CI, 0.02–0.26), and decreased length of hospital stay. RAM cases were also found to have longer operating times and higher costs, but there was no difference in terms of adverse outcomes. Compared to laparoscopic myomectomy, RAM showed no differences in terms of EBL, length of hospital stay, or adverse outcomes but did show a higher cost. Notably, the conversion to laparotomy (0.4% with traditional laparoscopy and 0.5% with RAM) was similar between groups with mean myoma diameter ranging from 5 to 9 cm. All but one study included had a mean myoma diameter of 5–7 cm.

While studies investigating RAM have demonstrated efficacy with a range of myoma sizes, there has not been an evaluation of the feasibility of RAM, specifically for large myomas, to date. While previous studies have evaluated surgical outcomes based on specimen weight during histopathologic evaluation, our study, in contrast, uses preoperative imaging to define treatment groups. We speculate that this approach is more useful when counseling patients preoperatively in order to determine the best candidates for RAM. Our study also highlights that surgeries are performed by a single surgeon which limits variation in surgical technique, thereby allowing the completion of challenging surgical cases, without adversely impacting surgical outcomes. It is well established that the challenges of RAM are related to not only myoma size, but also location and number. Specifically, intramural myomas can be associated with increased EBL and often require multiple layer closure to obtain adequate hemostasis. However, given that there was no difference between percentages of patients with primarily intramural myomas or >5 myomas in either study group, we feel that our conclusions regarding myoma size are not biased by the confounding factors such as location and number.

Our study is limited by its retrospective nature and sample size. Our lack of major adverse outcomes, namely, no conversions to laparotomy, may reflect the fact that technical skill associated with RAM has improved over the past several years. Further prospective studies are needed to validate our results and help delineate recommendations for size limits of myomas amenable to robotic surgery.

In conclusion, RAM may be a feasible surgical approach for patients with myomas ≥9 cm. Although RAM is associated with greater EBL and operative time, there was no change in major adverse outcomes. Furthermore, patients with large myomas undergoing RAM can be discharged on the same day after meeting immediate postoperative milestones. Practitioners should be aware of the alternative of a minimally invasive approach, especially RAM, to avoid the known morbidities associated with the open approach. Given the improved surgical dexterity associated with robot-assisted technology over traditional laparoscopy, the robot-assisted platform may enable safe removal of large myomas that may have previously been thought to only be amenable to an abdominal approach.

## Figures and Tables

**Table 1:** Demographic characteristics of leading myoma <9 cm versus ≥9 cm.

Parameter	Largest myoma <9 cm (*n* = 141)	Largest myoma ≥9 cm (*n* = 66)	*P*
Age	35.6 ± 5.8	36.9 ± 5.4	n.s.
Body mass index (kg/m^2^)	26.3 ± 6.1	24.7 ± 6.0	n.s.
Parity (median)	0	0	n.s.
% with previous abdominal surgery	53.5%	25.8%	<0.001
% with >5 myomas	34.8%	26.2%	n.s.
% intramural myoma	86.5%	86.2%	n.s.
Median specimen weight (gm)	106 ± 164.5	510 ± 379.5	<0.001

n.s.: not significant.

**Table 2:** Perioperative outcomes by leading myoma <9 cm versus ≥9 cm.

Parameter	Largest myoma <9 cm (*n* = 141)	Largest myoma ≥9 cm (*n* = 66)	*P*
Operative time (mins) [median (IQR)]	92 (70, 141)	130 (92, 188)	<0.001
EBL (mL) [median (IQR)]	25 (15, 50)	100 (25, 200)	<0.001
Mean duration of hospital stay (days)	0	0	n.s.

n.s.:  not significant.
